# Prescription medication use in the 10 years prior to diagnosis of young onset Alzheimer’s disease: a nationwide nested case-control study

**DOI:** 10.1186/s13195-024-01523-7

**Published:** 2024-07-05

**Authors:** Line Damsgaard, Janet Janbek, Thomas Munk Laursen, Karsten Vestergaard, Hanne Gottrup, Christina Jensen-Dahm, Gunhild Waldemar

**Affiliations:** 1 Danish Dementia Research Centre, Department of Neurology, Copenhagen University Hospital - Rigshospitalet, Copenhagen, Denmark; 2 National Centre for Register-based Research, Department of Economics and Business Economics, Aarhus University, Aarhus, Denmark; 3 Dementia Clinic, Department of Neurology, Aalborg University Hospital, Aalborg, Denmark; 4 Dementia Clinic, Department of Neurology, Aarhus University Hospital, Aarhus, Denmark; 5 Department of Clinical Medicine, University of Copenhagen, Copenhagen, Denmark

**Keywords:** Young onset dementia, Alzheimer’s disease, Medication, Early warning signs, Registry-based, Epidemiology

## Abstract

**Background:**

Patients with young onset Alzheimer’s disease (YOAD) face long diagnostic delays. Prescription medication use may provide insights into early signs and symptoms, which may help facilitate timely diagnosis.

**Methods:**

In a register-based nested case-control study, we examined medication use for everyone diagnosed with YOAD in a Danish memory clinic during 2016–2020 compared to cognitively healthy controls. Prescription medication use were grouped into 13 overall categories (*alimentary tract and metabolism, blood and blood forming organs, cardiovascular system, dermatologicals, genitourinary system and sex hormones, systemic hormonal preparations, antiinfectives for systemic use, antineoplastic and immunomodulating agents, musculo-skeletal system, nervous system, antiparasitic products, respiratory system*, and *sensory organs*). Further stratifications were done for predetermined subcategories with a use-prevalence of at least 5% in the study population. Conditional logistic regression produced odds ratios, which given the use of incidence-density matching is interpretable as incidence rate ratios (IRRs). The association between prescription medication use and subsequent YOAD diagnosis was examined in the entire 10-year study period and in three time-intervals.

**Results:**

The study included 1745 YOAD cases and 5235 controls. In the main analysis, several overall categories showed significant associations with YOAD in one or more time-intervals, namely *blood and blood forming organs* and *nervous system*. Prescription medication use in the *nervous system* category was increased for YOAD cases compared to controls already 10->5 years prior to diagnosis (IRR 1.17, 95% CI 1.05–1.31), increasing to 1.57 (95% CI 1.39–1.78) in the year preceding diagnosis. This was largely driven by antidepressant and antipsychotic use, and especially prominent for first-time users.

**Conclusions:**

In this study, medication use in several categories was associated with YOAD. Onset of treatment-requiring psychiatric symptoms such as depression or psychosis in mid-life may serve as potential early indicators of YOAD.

**Supplementary Information:**

The online version contains supplementary material available at 10.1186/s13195-024-01523-7.

## Background

Long diagnostic delays have been described in patients with young onset Alzheimer’s disease (YOAD) [1, 2], and a more comprehensive picture of the early warning signs of YOAD could be key in facilitating a timely diagnosis in this often overlooked patient group. In prior studies, we have shown that patients diagnosed with YOAD have increased healthcare utilization across all primary and secondary healthcare providers up to 10 years prior to dementia diagnosis, with a major surge in psychiatric healthcare use leading up to diagnosis [3]. Additionally, YOAD patients had increased hospital-diagnosed morbidity prior to diagnosis, especially psychiatric morbidity such as stress and depression [4]. Examining prescription medication use prior to YOAD may help us gather further insights into these early signs and may also serve as a proxy for symptoms and diseases not requiring hospital contact.

To our knowledge, no prior studies have examined medication use prior to a diagnosis of YOAD, though several studies have examined the association between specific types of medication and late onset Alzheimer’s disease (LOAD) or late onset all-cause dementia. These are often either risk factor studies or studies of agents that may be repurposed for treatment of dementia. For instance, such studies have concluded that exposure to strong anticholinergic drugs [5], benzodiazepines [6, 7], lithium [8], and antiepileptic drugs [9] is associated with an increased risk of (all-cause) dementia. However, given the age-related changes in morbidity burden and medication use, findings from studies involving older populations may not be directly applicable to individuals with YOAD.

The aim of this study was to explore whether individuals diagnosed with YOAD have an altered prescription medication use pattern in the 10-year lead-in to diagnosis, compared to cognitively healthy, matched adults.

## Methods

### Data sources

Cases were identified from the Danish Quality Database for Dementia (DanDem). All Danish healthcare facilities that accept referrals for diagnostic evaluation of dementia and cognitive impairment must enter information such as etiology, severity of the dementia syndrome at time of diagnosis (if applicable), and diagnostic investigations performed including results of selected cognitive tests, upon completion of diagnostic evaluation. DanDem contains information on all patients seen at Danish Memory clinics from establishment of the database in 2016 onwards. Information on medication use was drawn from the Danish National Prescription Registry (DNPrR), containing detailed information on all redeemed prescriptions since 1995 [10] using the World Health Organization’s Anatomical Therapeutic Chemical (ATC) code system [11]. The Danish National Patient Register (DNPR) and the Danish Psychiatric Central Research Register (DPCRR) were used for information on diagnoses given in hospitals, used in the selection/exclusion of cases and controls [12, 13]. Covariates were identified from the Population Education Register [14] and the Civil Registration System, with the latter also used for linkage between registers through each Danish citizen’s unique 10-digit personal identification number [15, 16].

### Study design and population

We conducted a retrospective nested case-control study following the approach of our previous studies on this population [3, 4]. Associations between prescription medication and YOAD were examined over a 10-year period. This period was chosen to account for the gradual onset and progression of pathology in Alzheimer’s disease [17], allowing for a comprehensive exploration of potential associations between medication use and disease onset.

#### Case definition

By convention, YOAD is defined as dementia due to Alzheimer’s disease (AD) with symptom onset before age 65 years. As the registries used do not contain information about time of symptom onset, we approximated this using date of diagnosis. As previous studies have shown an average of around 5 years from symptom onset to final diagnosis [1, 2], we included all patients diagnosed with mild cognitive impairment (MCI) or dementia due to AD before age 70 years, assuming symptom onset before age 65. Cases were identified from DanDem as individuals with a first diagnosis of MCI due to AD or dementia due to AD from start of register (2016) through 2020. Some cases also had a previous record related to dementia in other registers. For all cases, index time was set to time of diagnosis in DanDem (further details on choice of index date in supplementary methods 2.2.1.S).

#### Control definition

For each case, three age- and sex-matched controls were drawn from the full risk set (the entire Danish population). Incidence-density-sampling was used, as each control was at risk for YOAD at matching date. This allowed the odds ratios from the conditional logistic regression to be interpreted as incidence rate ratios (IRR) [18]. Exclusion criteria for controls were prior dementia (determined by dementia diagnosis in DNPR or DPCRR or redeemed prescription of antidementia medication in DNPrR) or memory clinic visits prior to index date (codes for identification of exclusion criteria: table S1).

#### Exclusion

Individuals with a primary/secondary diagnostic code indicating Down syndrome (ICD-8: 759.3, ICD-10: DQ90) and Mental Retardation (ICD-8: 311–315, ICD-10: DF70-DF79) were excluded from the study population. To ensure completeness of data, cases and controls must have lived in Denmark in the 10-year retrospective period. All exclusion criteria can be seen in table S1.

### Exposure and covariates

#### Exposure

Medication use was defined based on all redeemed prescriptions in the DNPrR in the 10-year study period. Medications were grouped according to (1) overall category (corresponding to ATC main groups, i.e., 1st digit of the ATC-code) and (2) subcategories grouping medications by indication (largely based on ATC therapeutic subgroups, but where relevant more detailed subcategories were chosen using three- or four-digit ATC-codes). Table S2 shows the definition of overall categories, subcategories, and corresponding ATC-codes. For chosen subcategories where < 5% of the study population had at least one redeemed prescription, these were included in the overall category’s “other” group for the regression analysis, as comparisons of medication use among fewer individuals would not yield meaningful results.

#### Covariates

Analyses were done in two models; one unadjusted, one adjusted for age, sex, highest attained educational level at age 40 years (or at time of diagnosis, whichever came first), and civil status at index date.

### Data analysis

#### Main analysis

For each overall category we used a conditional logistic regression model to investigate the association between having at least one redeemed prescription within the category and subsequent diagnosis of YOAD, yielding IRRs. Each matched set served as a stratum in the regression model. IRRs were calculated for each overall category for (a) the entire 10-year period, (b) the 10->5-year interval prior to diagnosis, (c) the 5->1 year interval prior to diagnosis, and (d) the ≤ 1-year interval prior to diagnosis to investigate latency between medication use and dementia diagnosis. As we have previously found psychiatric healthcare use and psychiatric morbidity to be significantly increased prior to YOAD [3, 4], we decided a-priori to examine medication in the *nervous system* subcategories in time-intervals. Associations between the medication use in the remaining subcategories and YOAD were assessed only in the entire 10-year period.

As any prior dementia medication resulted in exclusion for controls, ATC codes for antidementia medication (table S1) were omitted for cases in the conditional logistic regression analyses.

#### Sensitivity analysis

In a sensitivity analysis, we repeated the analysis of overall categories in the 10-year study period while grouping by disease severity to examine those with MCI/mild dementia or moderate/severe dementia, compared with their respective matched controls. Furthermore, this analysis was repeated stratifying on age (age < 55 years, age ≥ 55 years) and on sex, as well as omitting cases with MCI and their respective controls from the analyses. To ensure potential delays between actual and registered time of diagnosis did not impact results, we additionally conducted a sensitivity analysis repeating the analysis of overall categories in the < 1 year interval while omitting the 6 months immediately prior to index date.

In a post hoc analysis exploring associations between *nervous system* medication and YOAD, we limited the analyses to first-ever prescriptions in each time interval compared to never-users. Furthermore, we conducted a post-hoc analysis examining which subcategories where the main causes of the decreased IRRs in the < 1 year interval in relevant overall categories.

Results are presented with 95% confidence intervals (CI), corresponding to a 5% significance level. As the study is exploratory in nature we did not correct for multiple comparisons, though it is important to keep in mind that with numerous analyses, CIs cannot be interpreted as usual and should serve merely as a loose indicator. Results are presented unadjusted and adjusted for age, sex, highest attained educational level at age 40 years (or at time of diagnosis, whichever came first), and civil status at index date. All statistical analyses were performed with SAS 9.4 software using the PROC logistic procedure. This research project was approved by the Danish Data Protection Agency, Statistics Denmark, and the Danish Health Data Authority. Danish law does not require ethics committee approval or informed patient consent.

## Results

Out of 17,644 patients diagnosed with AD in DanDem between 2016 and 2020, 1827 were diagnosed before age 70 years. After exclusion criteria were applied, there were 1745 YOAD cases, who were matched with 5235 controls. The mean age at time of diagnosis was 64.5 years, and 42% were male (Table 1). At time of diagnosis, 5% of cases were categorized as having MCI, 58% as having mild dementia, and 37% with moderate/severe dementia due to AD. Cases and controls were similar in terms of educational attainment and civil status.

**Table 1:** Baseline characteristics of study population

	Cases *n* = 1745	Controls *n* = 5235
Age at index date, mean years (sd) [range]	64.5 (5.1) [35.8–69.9]	64.5 (5.1) [35.5–69.9]
Sex, male/female, *n* (%)	726 (42%) / 1019 (58%)	2178 (42%) / 3057 (58%)
Dementia syndrome severity at time of diagnosis, *n* (%)		
Mild cognitive impairment	84 (5%)	-
Mild dementia	1010 (58%)	-
Moderate dementia	530 (30%)	-
Severe dementia	121 (7%)	-
Cognitive examination scores at first visit^*^, mean (sd)		
MMSE	21.0 (5.5)	-
ACE	65.2 (15.4)	-
Educational attainment at age 40, *n* (%)		
Low	1184 (68%)	3629 (69%)
Medium	374 (21%)	1118 (21%)
High	104 (6%)	268 (5%)
Unknown	83 (5%)	220 (5%)
Civil status at index date, *n* (%)		
Married	1094 (63%)	3377 (65%)
Divorced	321 (18%)	869 (17%)
Widowed	133 (8%)	349 (7%)
Never married	177 (10%)	611 (12%)
Unknown	20 (1%)	29 (1%)
Individuals with ≥ 1 prescription within each overall category, *n* (%)		
A: Alimentary tract and metabolism	944 (54%)	2821 (54%)
B: Blood and blood forming organs	625 (36%)	1534 (29%)
C: Cardiovascular system	1145 (66%)	3360 (64%)
D: Dermatologicals	1044 (60%)	3142 (60%)
G: Genitourinary system and sex hormones	660 (39%)	1981 (38%)
H: Systemic hormonal preparations	413 (24%)	1303 (25%)
J: Antiinfectives for systemic use	1498 (86%)	4502 (86%)
I: Antineoplastic and immunomodulating agents	37 (2%)	140 (3%)
M: Musculo-skeletal system	1192 (68%)	3588 (69%)
N: Nervous system†	1310 (75%)	3564 (68%)
P: Antiparasitic products, insecticides, and repellents	371 (21%)	1171 (22%)
R: Respiratory system	951 (55%)	2842 (54%)
S: Sensory organs	892 (51%)	2668 (51%)

Abbreviations and additional information

^*^MMSE: Mini-Mental State Examination (reliable information for 1590 YOAD patients). ACE: Addenbrooke’s Cognitive Examination (reliable information for 1173 YOAD patients). † Not including antidementia medication

Sd: standard deviation

Note: Where percentages do not add up to 100%, this is due to rounding up/down

**Fig. 1:**
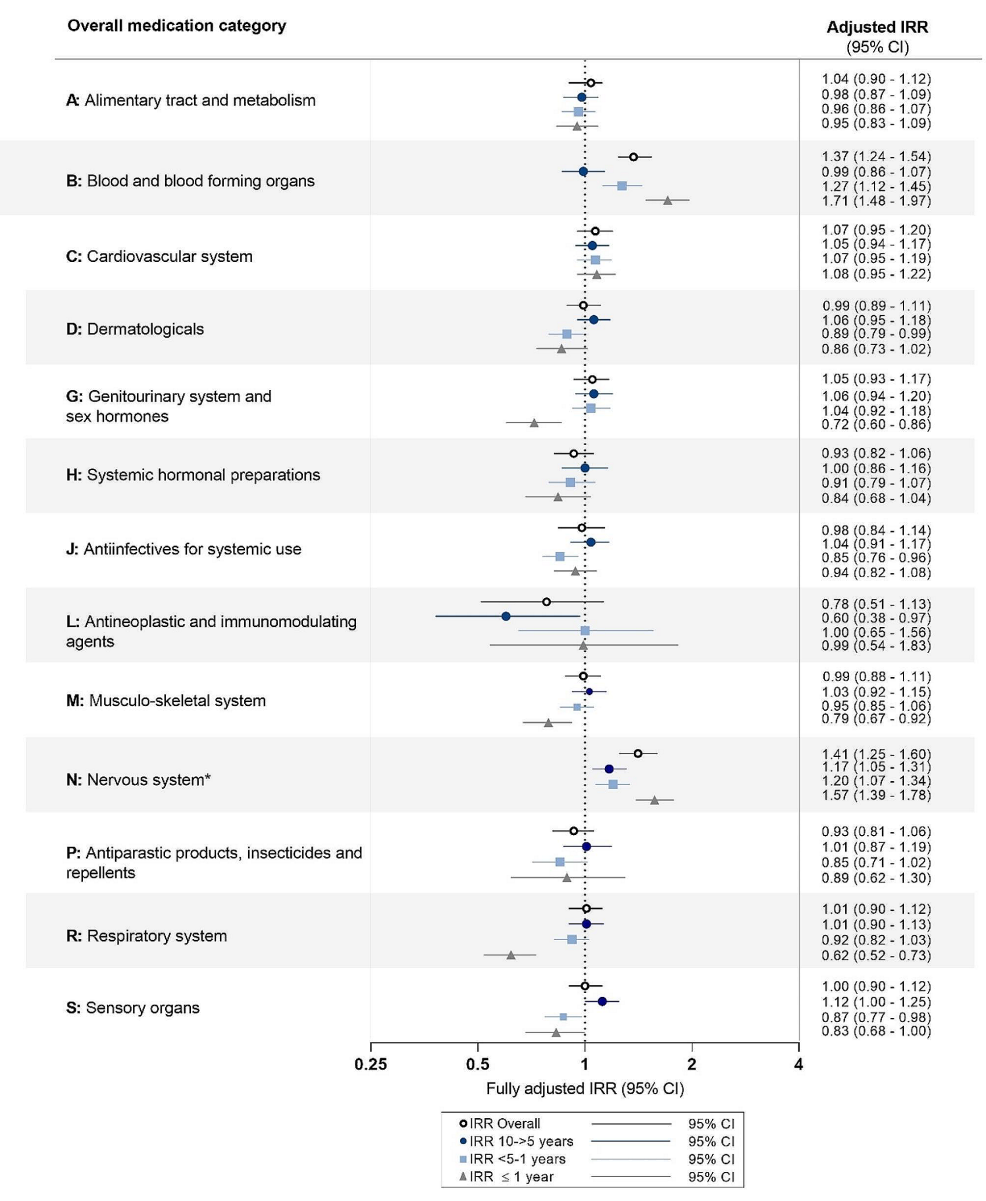
Incidence rate ratios by overall medication category in the 10-year study period and in time intervals. Incidence rate ratios (IRRs) for young onset Alzheimer’s disease are plotted by overall medication categories in the 10-year retrospective study period and in three time-intervals prior to diagnosis. Conditional logistic regression analyses produced odds ratios, which given the use of incidence-density-sampling is interpretable as IRRs. For the refence group (dementia-free controls), the IRR is equal to 1 (as indicated by the dotted vertical line). Error bars represent 95% confidence intervals (CI). The IRRs presented are adjusted for age, sex, highest attained educational level at age 40 years (or at time of diagnosis, whichever came first), and civil status at index date. Unadjusted estimates are presented in table S3. * Not including dementia medication (see ATC-codes used in table S3)

The proportion of cases and controls with a redeemed prescription within each overall category were nearly identical across all categories, except for medications in *blood and blood forming organs* (36% of cases, 29% of controls) and in *nervous system* (75% of cases, 68% of controls). For *blood and blood forming organs* medication, we found an IRR of 1.27 (95% CI 1.12–1.45) in the 5->1 years prior to diagnosis, increasing to 1.71 (95% CI 1.48–1.97) in the year immediately preceding YOAD diagnosis, compared to controls. *Nervous system* medications were increased with around 20% in both the 10->5- and 5->1-year periods, increasing to an IRR of 1.57 (95% CI 1.39–1.78) in the year before diagnosis (Fig. 1). The remaining overall categories had IRRs around 1, except *genitourinary system and sex hormones*, *musculo-skeletal system*, and *respiratory system* products, where IRRs were slightly decreased in the year prior to diagnosis. In a post-hoc analysis we examined the main drivers of the decreased IRRs; these were sex hormones, antiinflammatory and antirheumatic drugs, and all respiratory subcategories, respectively (data not shown).

**Fig. 2:**
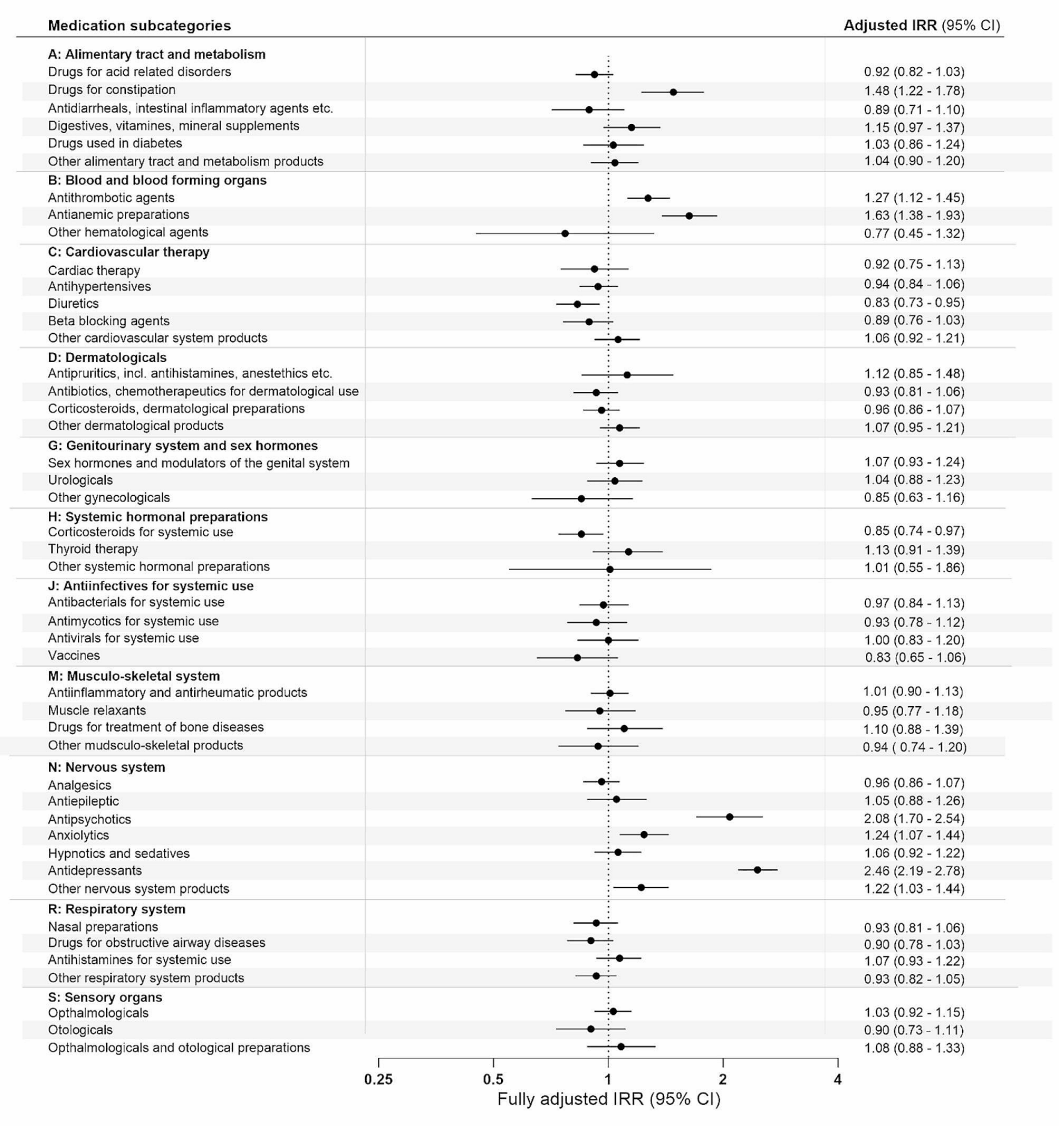
Incidence rate ratios by medication subcategories in the 10-year retrospective study period. Incidence rate ratios (IRRs) for young onset Alzheimer’s disease are plotted by medication subcategories in the 10-year retrospective study period. Conditional logistic regression analyses produced odds ratios, which given the use of incidence-density-sampling is interpretable as IRRs. For the refence group (dementia-free controls), the IRR is equal to 1 (as indicated by the dotted vertical line). Error bars represent 95% confidence intervals (CI). The IRRs presented are adjusted for age, sex, highest attained educational level at age 40 years (or at time of diagnosis, whichever came first), and civil status at index date. Unadjusted estimates are presented in table S4

**Fig. 3:**
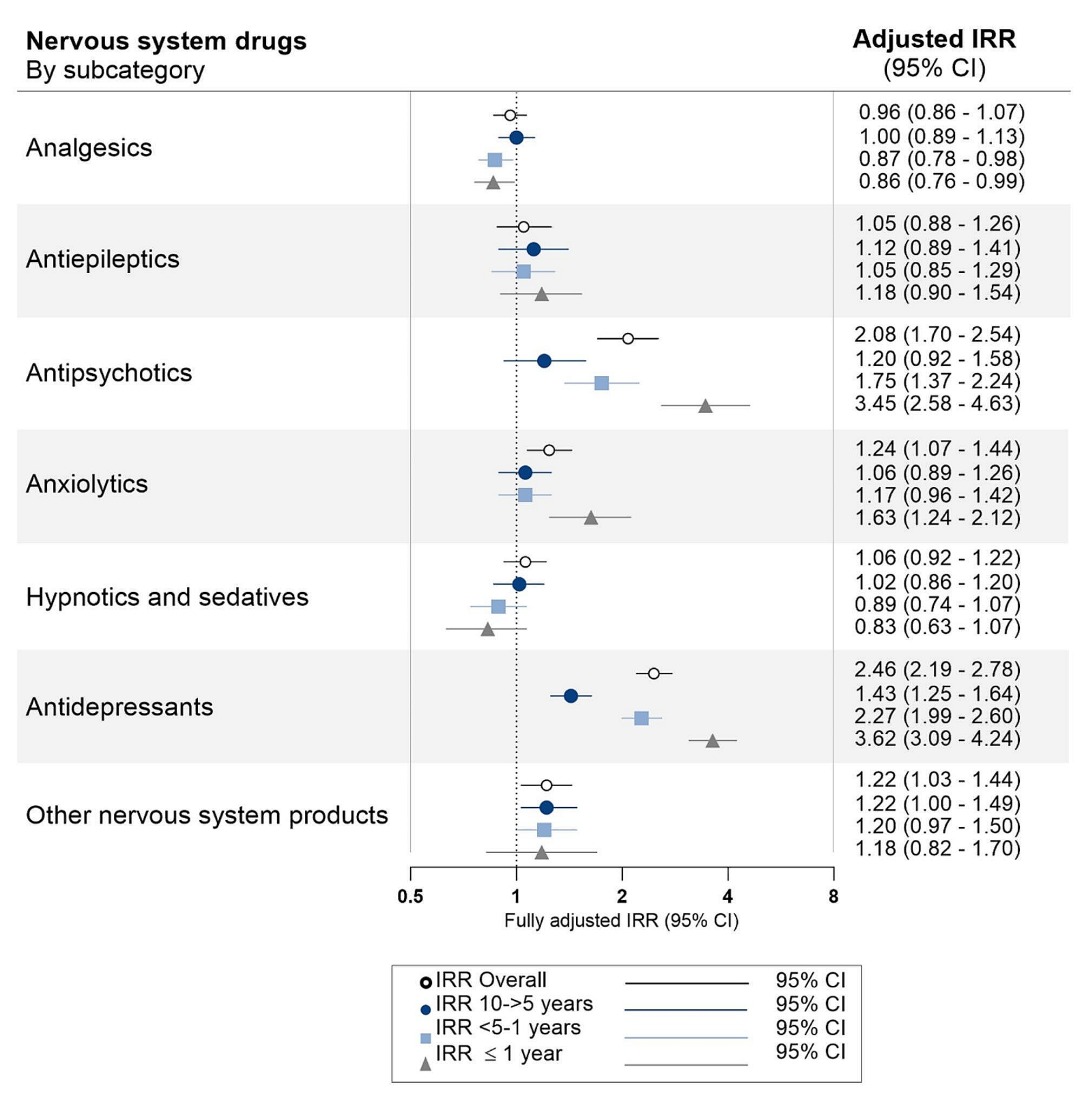
Incidence rate ratios by nervous system subcategories in the 10-year study period and in time intervals. Incidence rate ratios (IRRs) for young onset Alzheimer’s disease are plotted by *nervous system* subcategories in the 10-year retrospective study period and in three time-intervals prior to diagnosis. Conditional logistic regression analyses produced odds ratios, which given the use of incidence-density-sampling is interpretable as IRRs. For the refence group (dementia-free controls), the IRR is equal to 1 (as indicated by the dotted vertical line). Error bars represent 95% confidence intervals (CI). The IRRs presented are adjusted for age, sex, highest attained educational level at age 40 years (or at time of diagnosis, whichever came first), and civil status at index date. Unadjusted estimates are presented in table S5

Figure 2 shows associations between medication subcategories (with a use-prevalence of at least 5%) and YOAD diagnosis. Of the 46 subcategories examined, seven had significantly increased IRRs. The most notable increases were found for use of antidepressants, antipsychotics, antianemic preparations, antithrombotic preparations, and drugs for constipation, with smaller increases for anxiolytics and other nervous system products. Looking at *nervous system* drugs in time-intervals (Fig. 3), the increased use of antidepressants became apparent already 10->5-years prior to diagnosis (IRR 1.43, 95% CI 1.25–1.64) and increased steadily as index date approached. Antipsychotic use was increased from 5->1-year prior to diagnosis, while the use of anxiolytics only showed an increased IRR in the year prior to diagnosis. In a post-hoc analysis examining only first-ever prescriptions of *nervous system* drugs compared to never-users, these effects were reinforced with IRRs over 10 found for both antipsychotic- and antidepressant use in the year preceding diagnosis (figure S1).

In a sensitivity analysis stratifying by dementia syndrome severity at index date, we divided the study population in two groups: cases with MCI/mild dementia and their controls, and cases with moderate/severe dementia and their controls. Results were similar to those found in the main analysis, though the magnitude of the association between the use of *nervous system* medication and YOAD differed slightly (figure S2). Furthermore, we stratified the study population by sex (figure S3) and age (figure S4), which yielded results similar to those found in the main analysis, as did omitting cases with MCI (table S10) and prescription medication use in the six months prior to index date (table S11). Unadjusted estimates are presented for all analyses in tables S3-S11. Adjustment generally did not impact the estimates.

## Discussion

In this nationwide nested case-control study, we found notable variations in medication use between individuals with a YOAD diagnosis and those without. Particularly noteworthy were differences in utilization of *nervous system* medications. Those with YOAD had a 17% increased use of medications within the *nervous system* category already in the 10->5 years leading up to index date, increasing to 57% in the year immediately prior. This was largely driven by the use of antidepressants and antipsychotics, and the difference between cases and controls was more pronounced when comparing those with a first-ever prescription to never-users. Hence, initiation of these medications in mid-life could potentially serve as an indicator of YOAD. We note, though, that all confidence intervals (and hence, significance) should be interpreted with caution given the number of associations examined.

To our knowledge, this is the first study exploring medication use prior to YOAD, though a few studies have broadly examined medication use prior to all-cause dementia or LOAD. One such study showed associations between Sertraline, Escitalopram, and Mirtazapine (all medications used to treat depression) and AD with hazard ratios around 2-3 [19], while a study specifically investigating antidepressants found a slight increase in the rate of antidepressant use among AD patients already 9 years before diagnosis [20]. This aligns with our findings, implying an association between depression and AD, perhaps showing that depression is part of the AD prodrome both in YOAD and LOAD, or that early dementia symptoms such as apathy, withdrawal, and changes in mood and behaviour, are misinterpreted as symptoms of depression. This corresponds with our previous findings of increased psychiatric healthcare utilization [3] and depression diagnoses [4] in the 10 years prior to YOAD diagnosis, rising as time of diagnosis approaches. This could reflect either a prodromal increased susceptibility to depression among those with AD pathology but as yet without dementia symptoms, or dementia symptoms misinterpreted as depression. It may also reflect depression occurring comorbidly with YOAD or depression unrelated to dementia symptoms and pathology. Another study found that those who experienced onset of psychotic symptoms after the age of 50 years had a greater risk for cognitive impairment. This was particularly pronounced in carriers of the *APOE* ε4 allele [21], an important genetic risk factor in YOAD. In line with these findings, the present study found a significant association between first-time use of antipsychotic medication and YOAD as much as five years prior to dementia diagnosis. Our findings thus support their conclusion; that onset of psychotic symptoms in later life could warrant cognitive evaluation in a memory clinic.

In addition to our findings for medication in the *nervous system* category, another key finding was an increased IRR for the overall category *blood and blood forming organs* medications from <5 years prior to YOAD diagnosis. Subcategories with increased IRR within this chapter were antithrombotic agents and antianemic preparations. Vascular disease is known to increase the risk of LOAD [22], and oral anticoagulant use have been shown to be higher among those who are later diagnosed with AD [23]. Our findings also imply an association between vascular diseases and YOAD, perhaps indicating that vascular risk factors may play a role also in YOAD. However, the present study did not aim to assess risk, for which measures to mitigate reverse causation etc. would be needed.

Aside from drugs in the two discussed categories (*nervous system* and *blood and blood forming organs*), we also noted significant associations in our study of either increased or decreased IRRs for other medications. We discuss these findings in the following but note that given the number of associations examined, marginally significant results should be interpreted cautiously.

A significantly increased IRR was found for the subcategory drugs for constipation. This mirrors findings from two large studies where hazard ratios of around 1.3–2.3 were found for AD following prior laxative use [24, 25]. Similar associations have been found between laxative use and all-cause dementia [26]. Constipation is a well-known early symptom in dementia with Lewy bodies and Parkinson’s disease [27], and further research could investigate whether it may be an early marker of YOAD as well or perhaps an expression of dual pathology of AD and dementia with Lewy bodies.

In the following we address findings related to decreased IRRs. Within the <1 year period leading up to index date, medication in the category *genitourinary system and sex hormones* had an IRR of 0.72, with the decrease largely driven by the subcategory sex hormones. The most commonly used form of medication within this subcategory was estradiol vaginal tablets. A nationwide Danish cohort study examining use of vaginal estrogen found no association with AD, with the authors suggesting impaired ability to act on urogenital symptoms and diminished compliance with already initiated therapy just prior to diagnosis [28], which could also be the explanation for our findings. Another decreased IRR in the < 1 year interval was found for *musculo-skeletal system drugs*, driven by antiinflammatory and antirheumatic drugs. The role of inflammation in AD pathology has been a topic of scientific interest in recent years [29], with some studies suggesting antiinflammatory drugs may lower the risk of dementia [30]. However, our study is not suitably designed to determine risk factors, and given the proximity to YOAD diagnosis, this decreased IRR in the year prior to diagnosis is likely explained by other factors and potential bias such as reverse causality. Lastly, in the analysis of subcategories we found a decreased IRR for corticosteroids for systemic use, albeit borderline significant. While Prednisone was found to be associated with lower rates of dementia in another study [19], mechanisms behind this possible association is unclear, and further research is warranted.

### Limitations

Our study had several limitations. First, although healthcare is free of charge in Denmark, there is (some) out-of-pocket payment for prescription medication, perhaps favouring more resourceful individuals among medication users. Second, in viewing medications as a proxy measure of symptoms and diseases, dual uses of some drugs may cause us to miss valuable information – for example, some drugs registered in the ATC-system as antiepileptic drugs are also commonly used for psychiatric disorders, leading to a potential under-estimation of the use of drugs with psychiatric indication. Likewise, some drugs registered for psychiatric disorders are sometimes used for other indications (the use of tricyclic antidepressants for neuropathic pain, for example), leading to a potential over-estimation. Third, some of the analyses – especially those of first-ever prescriptions – are prone to detection bias; those starting a new medication will likely have been seen by a doctor, increasing the likelihood of detection of cognitive symptoms. However, looking for example at antidepressant use, we find a near four-fold use among YOAD cases in the main analysis, and a near 13-fold first-ever use. While detection bias may be a contributing factor, we find it unlikely that this alone can entirely explain the large difference between ever-users and first-ever users. Fourth, as the present study is exploratory in nature, causal inferences cannot be made. Consequently, we encourage future research to investigate the potential causal roles of the associations found while employing the appropriate methodologies to assess causality.

A major strength of our study is the use of the high-quality Danish registers. Using the quality registry DanDem for case finding allowed us to establish a large case cohort while ensuring that diagnosis of YOAD was made by specialists in memory clinic settings, and to include detailed variables about disease severity, test scores, etc. The use of nationwide healthcare registers to assess covariates and exposure information allowed for virtually full data coverage, limiting selection bias. We have previously examined morbidity prior to a diagnosis of YOAD, however due to data availability it was only possible to examine hospital-diagnosed morbidity. Looking at prescription medication allows us to supplement the knowledge gained previously by adding a proxy for symptoms not necessarily necessitating a hospital contact. Future research should aim at exploring the intricate interplay between different medication categories, morbidity, and concurrent morbidities or medication use.

### Conclusions

The key takeaway from our study is that the onset of depression or psychotic symptoms in mid-life may serve as potential early indicators of YOAD. This is consistent with our previous findings of increased psychiatric healthcare utilization and morbidity in this patient group [3, 4]. These findings underscore the importance of considering mental health symptoms as a potential part of the prodromal phase of YOAD, providing valuable insights for early detection as awareness of early symptoms may help facilitate a timely diagnosis. Future studies could examine the predictive value of these psychiatric symptoms in identifying individuals with undiagnosed YOAD.

### Electronic supplementary material

Below is the link to the electronic supplementary material.

Supplementary Material 1

## Data Availability

All data used in this study are derived from the Danish National and Public Health registries. These data are collected and stored by the relevant authorities and cannot be made public or accessed by unauthorized parties. Access to such data is given via standard rules and regulations of data access outlined by the Danish Data Protection Agency and Danish Health Data Authority.

## Abbreviations

YOAD: Young onset Alzheimer’s disease
LOAD: Late-onset Alzheimer’s disease
DanDem: The Danish Quality Database for Dementia
DNPrR: The Danish National Prescription Registry
ATC: Anatomical Therapeutic Chemical
DNPR: The Danish National Patient Register
DPCRR: The Danish Psychiatric Central Research Register
MCI: Mild cognitive impairment
AD: Alzheimer’s disease
IRR: Incidence rate ratio
CI: Confidence intervals
