# Supplementary material for: Prescription medication use in the 10 years prior to diagnosis of young onset Alzheimer’s disease: a nationwide nested case-control study
Source: Alzheimers Res Ther. 2024 Jul 5;16:150. doi: 10.1186/s13195-024-01523-7 (PMC11225233; doi:10.1186/s13195-024-01523-7)
Supplement: Supplementary file 1 — Supplementary Material 1 [file 13195_2024_1523_MOESM1_ESM.pdf]

<u>**Supplementary - Medication use in the 10 years prior to diagnosis of young onset Alzheimer's disease**</u>

Damsgaard L, Janbek J, Laursen TM, Vestergaard K, Gottrup G, Jensen-Dahm C, Waldemar G.

<u>**Content:**</u>

Supplementary methods:

2.2.1.S Case definition: index date

Supplementary tables:

Table S1. Exclusion criteria

Table S2. Overall categories, subcategories, and corresponding ATC codes

Table S3. Incidence rate ratios for medication use in overall categories, overall and in time-intervals

Table S4. Incidence rate ratios for medication use in subcategories

Table S5. Incidence rate ratios for medication use in *nervous system* subcategory in time-intervals

Table S6. Post-hoc analysis - Incidence rate ratios for medication use in *nervous system* subcategory (first prescriptions only)

Table S7. Sensitivity analysis by dementia syndrome severity at time of diagnosis – incidence rate ratios by overall categories

Table S8. Sensitivity analysis by age at time of diagnosis – incidence rate ratios by overall categories

Table S9. Sensitivity analysis by sex – incidence rate ratios by overall categories

Table S10. Sensitivity analysis, omitting MCI

Table S11. Sensitivity analysis, omitting prescription medication use 6 months prior to index date

Supplementary figures:

Figure S1. Incidence rate ratios for medication use in *nervous system* subcategory in time-intervals (first prescriptions only)

Figure S2. Sensitivity analysis by dementia syndrome severity at time of diagnosis – incidence rate ratios by overall categories

Figure S3. Sensitivity analysis by age at time of diagnosis – incidence rate ratios by overall categories

Figure S4. Sensitivity analysis by sex – incidence rate ratios by overall categories

Supplementary methods:

2.2.1.S Case definition: index date

For the present study, we chose date of diagnosis in DanDem as index date. Of cases, 64% had a diagnostic code of dementia or mild cognitive impairment (ICD-8 or ICD-10 codes listed in table S1) in DNPR/DPCRR prior to date of diagnosis in DanDem, with a median time of 93 days between the two. In some of the Danish memory clinics, patients are routinely coded by administrative personnel with an unspecified dementia-code upon being referred to the memory clinic prior to diagnostic assessment, and some are given a dementia-code in the registers by the referring department without cognitive evaluation; therefore, the diagnostic code is often given before the actual time of diagnosis. The median time of 93 days corresponds well with usual time frame from first referral to diagnosis in the memory clinics. Furthermore, prior research has shown a high validity of dementia-codes for individuals with late onset dementia in the Danish registers, though validity of dementia subtypes is generally low[1]. For patients with young onset dementia, though, there is a low validity of dementia diagnosis in DNPR/DPCRR[2,3]. For these reasons, index date from DanDem was chosen for all cases, as this represents the most reliable source of information on the date of AD diagnosis.

References:

1. Phung TKT, Andersen BB, Høgh P, Kessing LV, Mortensen PB, Waldemar G. Validity of dementia diagnoses in the Danish hospital registers. *Dement Geriatr Cogn Disord*. 2007;24(3):220-228. doi:10.1159/000107084
2. Salem LC, Andersen BB, Nielsen TR, et al. Overdiagnosis of dementia in young patients-a nationwide register-based study. *Dement Geriatr Cogn Disord*. 2013;34(5-6):292-299. doi:10.1159/000345485
3. Salem LC, Andersen BB, Nielsen TR, Stokholm J, Jørgensen MB, Waldemar G. Inadequate Diagnostic Evaluation in Young Patients Registered with a Diagnosis of Dementia: A Nationwide Register-Based Study. *Dement Geriatr Cogn Dis Extra*. 2014;4(1):31-44. doi:10.1159/000358050

Supplementary tables

Table S1. Exclusion criteria

	Cases	Controls
**Developmental disorders and mental retardation**<br>ICD-8: 311-315, 759.3, ICD-10: DF70-DF79, DQ90	X	X
**Not living in Denmark in 10-year retrospective period**	X	X
**Dementia or mild cognitive impairment diagnosis**<br>ICD-8: 290.09-11, 290.18-19, 293.09-19, ICD 10: F00.0-00.9, F01.0-01.9, F02.0-F02.8, F03.9, F04.0-F04.9, F06.7 G30.0-G30.9, G31.0A, G31.0B, G31.8, G31.8E, G31.9		X
**Dementia medication**<br>ATC code N06DA02-4, N06DX01	*	X
**Entry in DanDem**		X

***** These ATC codes registered in the Danish National Prescription Registry are omitted in the analyses.
ICD: International classification of diseases, ATC: Anatomical therapeutic chemical code, DanDem: Danish Quality Database for Dementia

Table S2. Overall categories, subcategories, and corresponding ATC codes

Overall category	ATC code	Subcategory	ATC codes
Alimentary tract and metabolism	A	Drugs for acid related disorders	A02
		*Antiemetics and antinauseants**	*A04**
		Drugs for constipation	A06
		Antidiarrheals, intestinal antiinflammatory/antiinfective agents	A07
		*Antiobesity preparations, excl. diet products**	*A08**
		Digestives, vitamins and mineral supplements, tonics	A09, A11, A12, A13
		Drugs used in diabetes	A10
		Other alimentary tract and metabolism products	A01, A03, A04, A05, A08, A14, A15, A16
Blood and blood forming organs	B	Antithrombotic agents	B01
		*Antihemorrhagics**	*B02**
		Antianemic preparations	B03
		Other hematological agents	B02, B05, B06
Cardiovascular system	C	Cardiac therapy	C01
		Antihypertensives	C02, C08, C09
		Diuretics	C03
		Beta blocking agents	C07
		Other cardiovascular system products	C04, C05
Dermatologicals	D	Antipruritics, incl. antihistamines, anesthetics, etc., antipsoriasis	D04, D05
		Antibiotics and chemotherapeutics for dermatological use	D06
		Corticosteroids, dermatological preparations	D07
		*Anti-acne preparations**	*D10**
		Other dermatological products	D01, D02, D03, D08, D09, D10, D11
Genito urinary system and sex hormones	G	*Gynecological antiinfectives and antiseptics**	*G01**
		Sex hormones and modulators of the genital system	G03
		Urologicals	G04
		Other gynecologicals	G01, G02
Systemic hormonal preparations, excluding sex hormones and insulin	H	*Pituitary and hypothalamic hormones and analogues**	*H01**
		Corticosteroids for systemic use	H02
		Thyroid therapy	H03
		*Pancreatic hormones**	*H04**
		*Calcium homeostasis**	*H05**
		Other systemic hormonal preparations	H01, H04, H05
Antiinfectives for systemic use	J	Antibacterials for systemic use	J01
		Antimycotics for systemic use	J02
		Antivirals for systemic use	J05
		Vaccines	J07
		Other antiinfective products†	*J04, J06†*
Antineoplastic and immunomodulating agents	L	*Antieneoplastic agents‡*	*L01‡*
		Endocrine therapy‡	*L02‡*

		Immunostimulants‡	*L03‡*
		Immunosuppressants‡	*L04‡*
Musculo-skeletal system	M	Antiinflammatory and antirheumatic products	M01
		*Muscle relaxants**	*M03**
		*Antigout preparations**	*M04**
		Drugs for treatment of bone diseases	M05
		Other musculo-skeletal products	M02, M03, M04, M09
Nervous system	N	Analgesics	N02
		Antiepileptics	N03
		*Anti-parkinson drugs**	*N04**
		Antipsychotics	N05A
		Anxiolytics	N05B
		Hypnotics and sedatives	N05C
		Antidepressants	N06A, N06B
		Other nervous system products	N01, N04, N07, N06C
Antiparasitic products, insecticides, and repellents	P	*None chosen ‡*	*‡*
Respiratory system	R	Nasal preparations	R01
		*Throat preparations**	*R02*
		Drugs for obstructive airway diseases	R03
		Antihistamines for systemic use	R06
		Other respiratory system products	R02, R05, R07
Sensory organs	S	Ophthalmologicals	S01
		Otologicals	S02
		Ophthalmologicals and otological preparations	S03

Subcategories in cursive were not individually analyzed:
* These categories were proposed as individual categories, but due to <5% of the study population having at least one redeemed prescription during the study period, these subcategories were combined with the "other" category within that overall category.
† As there were <5% of the study population with a redeemed prescription in the proposed "other" category, this was omitted from the analysis.
‡ Within the overall category Antineoplastic and immunomodulating agents, there were no proposed subcategories where >5% of the study population had a redeemed prescription. Within the Antiparasitic products, insecticides and repellents no relevant subcategories were proposed. Therefore, there were no relevant subcategories to examine for these two overall categories.
The ATC main group "Various" were omitted from all analyses due to low number of observations.

Table S3. Incidence rate ratios for medication use in overall categories, overall and in time-intervals

Overall category	Time-interval	Unadjusted		Adjusted	
		IRR	95% CI	IRR	95% CI
**Alimentary tract and metabolism**	**Overall**	1.01	0.90-1.13	1.04	0.90-1.12
**A**	10->5 years prior to index date	0.98	0.88-1.10	0.98	0.87-1.09
	5->1 years prior to index date	0.96	0.86-1.07	0.96	0.86-1.07
	≤1 year prior to index date	0.95	0.83-1.09	0.95	0.83-1.09
**Blood and blood forming organs**	**Overall**	1.36	1.21-1.53	1.37	1.24-1.54
B	10->5 years prior to index date	0.99	0.86-1.14	0.99	0.86-1.14
	5->1 years prior to index date	1.28	1.12-1.45	1.27	1.12-1.45
	≤1 year prior to index date	1.70	1.48-1.96	1.71	1.48-1.97
**Cardiovascular system**	**Overall**	1.07	0.95-1.20	1.07	0.95-1.20
**C**	10->5 years prior to index date	1.04	0.93-1.16	1.05	0.94-1.17
	5->1 years prior to index date	1.06	0.95-1.18	1.07	0.95-1.19
	≤1 year prior to index date	1.07	0.94-1.21	1.08	0.95-1.22
**Dermatologicals**	**Overall**	0.99	0.89-1.11	0.99	0.89-1.11
D	10->5 years prior to index date	1.06	0.95-1.18	1.06	0.95-1.18
	5->1 years prior to index date	0.89	0.80-0.99	0.89	0.79-0.99
	≤1 year prior to index date	0.87	0.74-1.03	0.86	0.73-1.02
**Genito urinary system and sex hormones**	**Overall**	1.05	0.94-1.18	1.05	0.93-1.17
G	10->5 years prior to index date	1.06	0.94-1.20	1.06	0.94-1.20
	5->1 years prior to index date	1.05	0.93-1.18	1.04	0.92-1.18
	≤1 year prior to index date	0.72	0.61-0.86	0.72	0.60-0.86
**Systemic hormonal preparations, excluding sex hor-mones and insulin**	**Overall**	0.93	0.82-1.06	0.93	0.82-1.06
	10->5 years prior to index date	1.00	0.87-1.17	1.00	0.86-1.16
H	5->1 years prior to index date	0.93	0.78-1.08	0.91	0.79-1.07
	≤1 year prior to index date	0.84	0.68-1.05	0.84	0.68-1.04
**Antiinfectives for systemic use**	**Overall**	0.99	0.84-1.16	0.98	0.84-1.14
J	10->5 years prior to index date	1.04	0.92-1.18	1.04	0.91-1.17
	5->1 years prior to index date	0.86	0.77-0.96	0.85	0.76-0.96
	≤1 year prior to index date	0.95	0.82-1.09	0.94	0.82-1.08
**Antineoplastic and immunomodulating agents**	**Overall**	0.79	0.55-1.14	0.78	0.51-1.13
L	10->5 years prior to index date	0.60	0.38-0.96	0.60	0.38-0.97
	5->1 years prior to index date	1.01	0.65-1.57	1.00	0.65-1.56
	≤1 year prior to index date	1.00	0.54-1.84	0.99	0.54.1.83
**Musculo-skeletal system**	**Overall**	0.99	0.88-1.11	0.99	0.88-1.11
M	10->5 years prior to index date	1.03	0.92-1.14	1.03	0.92-1.15
	5->1 years prior to index date	0.95	0.85-1.06	0.95	0.85-1.06
	≤1 year prior to index date	0.79	0.68-0.93	0.79	0.67-0.92

**Nervous system***	**Overall**	1.42	1.25-1.60	1.41	1.25-1.60
N	10->5 years prior to index date	1.18	1.06-1.31	1.17	1.05-1.31
	5->1 years prior to index date	1.21	1.08-1.35	1.20	1.07-1.34
	≤1 year prior to index date	1.58	1.40-1.79	1.57	1.39-1.78
**Antiparasitic products, insecticides, and repellents**	**Overall**	0.94	0.82-1.07	0.93	0.81-1.06
P	10->5 years prior to index date	1.02	0.87-1.19	1.01	0.87-1.19
	5->1 years prior to index date	0.86	0.72-1.03	0.85	0.71-1.02
	≤1 year prior to index date	0.90	0.62-1.31	0.89	0.62-1.30
**Respiratory system**	**Overall**	1.01	0.90-1.13	1.01	0.90-1.12
R	10->5 years prior to index date	1.01	0.90-1.13	1.01	0.90-1.13
	5->1 years prior to index date	0.92	0.82-1.03	0.92	0.82-1.03
	≤1 year prior to index date	0.62	0.53-0.73	0.62	0.52-0.73
**Sensory organs**	**Overall**	1.01	0.90-1.12	1.00	0.90-1-12
S	10->5 years prior to index date	1.12	1.00-1.25	1.12	1.00-1.25
	5->1 years prior to index date	0.87	0.77-0.98	0.87	0.77-0.98
	≤1 year prior to index date	0.84	0.69-1.01	0.83	0.68-1.00

*Dementia medication (table S1) is omitted in the conditional logistic regression

Table S4. Incidence rate ratios for medication use in subcategories (nervous system products presented in table S5)

Overall category	n	Subcategory	Unadjusted		Adjusted	
			IRR	95% CI	IRR	95% CI
**Alimentary tract and metabolism**	2670	Drugs for acid related disorders	0.93	0.83-1.04	0.92	0.82-1.03
A	575	Drugs for constipation	1.50	1.24-1.80	1.48	1.22-1.78
	496	Antidiarrheals, intestinal antiinflammatory/antiinfective agents	0.89	0.72-1.10	0.89	0.71-1.10
	749	Digestives, vitamins and mineral supplements, tonics	1.16	0.98-1.38	1.15	0.97-1.37
	678	Drugs used in diabetes	1.03	0.86-1.34	1.03	0.86-1.24
	1172	Other alimentary tract and metabolism products	1.04	0.90-1.20	1.04	0.90-1.20
**Blood and blood forming organs**	1668	Antithrombotic agents	1.27	1.12-1.44	1.27	1.12-1.45
B	701	Antianemic preparations	1.64	1.39-1.94	1.63	1.38-1.93
	82	Other hematological agents	0.78	0.46-1.34	0.77	0.45-1.32
**Cardiovascular system**	564	Cardiac therapy	0.92	0.75-1.13	0.92	0.75-1.13
C	2687	Antihypertensives	0.94	0.84-1.05	0.94	0.84-1.06
	1642	Diuretics	0.84	0.73-0.95	0.83	0.73-0.95
	1345	Beta blocking agents	0.89	0.77-1.02	0.89	0.76-1.03
	1290	Other cardiovascular system products	1.05	0.92-1.21	1.06	0.92-1.21
**Dermatologicals**	266	Antipruritics, incl. antihistamines, anesthetics, etc., antipsoriasis	1.12	0.85-1.48	1.12	0.85-1.48
D	1427	Antibiotics and chemotherapeutics for dermatological use	0.94	0.82-1.08	0.93	0.81-1.06
	3004	Corticosteroids, dermatological preparations	0.96	0.86-1.07	0.96	0.86-1.07
	2140	Other dermatological products	1.08	0.96-1.21	1.07	0.95-1.21
**Genito urinary system and sex hormones**	1652	Sex hormones and modulators of the genital system	1.07	0.92-1.23	1.07	0.93-1.24
G	1011	Urologicals	1.06	0.90-1.25	1.04	0.88-1.23
	252	Other gynecologicals	0.85	0.62-1.15	0.85	0.63-1.16
**Systemic hormonal preparations, excluding sex**	1300	Corticosteroids for systemic use	0.86	0.74-0.99	0.85	0.74-0.97
**hormones and insulin**	501	Thyroid therapy	1.13	0.92-1.40	1.13	0.91-1.39
H	56	Other systemic hormonal preparations	1.00	0.54-1.84	1.01	0.55-1.86
**Antiinfectives for systemic use**	5889	Antibacterials for systemic use	0.98	0.84-1.14	0.97	0.84-1.13
J	769	Antimycotics for systemic use	0.94	0.79-1.13	0.93	0.78-1.12
	678	Antivirals for systemic use	1.00	0.83-1.20	1.00	0.83-1.20
	385	Vaccines	0.84	0.66-1.08	0.83	0.65-1.06
**Musculo-skeletal system**	4578	Antiinflammatory and antirheumatic products	1.01	0.90-1.13	1.01	0.90-1.13
M	493	Muscle relaxants	0.96	0.78-1.19	0.95	0.77-1.18
	414	Drugs for treatment of bone diseases	1.11	0.88-1.39	1.10	0.88-1.39
	375	Other musculo-skeletal products	0.94	0.74-1.21	0.94	0.74-1.20
**Respiratory system**	1541	Nasal preparations	0.93	0.82-1.06	0.93	0.81-1.06
R	1424	Drugs for obstructive airway diseases	0.90	0.79-1.04	0.90	0.78-1.03
	1406	Antihistamines for systemic use	1.07	0.94-1.22	1.07	0.93-1.22
	1884	Other respiratory system products	0.93	0.82-1.05	0.93	0.82-1.05
**Sensory organs**	3210	Ophthalmologicals	1.04	0.93-1.16	1.03	0.92-1.15
S	533	Otologicals	0.90	0.73-1.11	0.90	0.73-1.11
	518	Ophthalmologicals and otological preparations	1.08	0.88-1.32	1.08	0.88-1.33

Table S5. Incidence rate ratios for medication use in *nervous system* subcategory in time-intervals

Nervous system subcategory	n	Time-interval	Unadjusted IRR	Unadjusted 95% CI	Adjusted IRR	Adjusted 95% CI
**Analgesics**	4080	**Overall**	0.96	0.86-1.08	0.96	0.86-1.07
		10->5 years prior to index date	1.01	0.90-1.13	1.00	0.89-1.13
		5->1 years prior to index date	0.88	0.79-0.98	0.87	0.78-0.98
		≤1 year prior to index date	0.88	0.77-1.00	0.86	0.76-0.99
**Antiepileptics**	728	**Overall**	1.06	0.89-1.26	1.05	0.88-1.26
		10->5 years prior to index date	1.12	0.89-1.41	1.12	0.89-1.41
		5->1 years prior to index date	1.06	0.86-1.31	1.05	0.85-1.29
		≤1 year prior to index date	1.20	0.92-1.57	1.18	0.90-1.54
**Antipsychotics**	448	**Overall**	2.10	1.72-2.55	2.08	1.70-2.54
		10->5 years prior to index date	1.21	0.92-1.58	1.20	0.92-1.58
		5->1 years prior to index date	1.78	1.40-2.28	1.75	1.37-2.24
		≤1 year prior to index date	3.51	2.63-4.69	3.45	2.58-4.63
**Anxiolytics**	1003	**Overall**	1.26	1.08-1.46	1.24	1.07-1.44
		10->5 years prior to index date	1.08	0.91-1.28	1.06	0.89-1.26
		5->1 years prior to index date	1.19	0.98-1.44	1.17	0.96-1.42
		≤1 year prior to index date	1.63	1.25-2.13	1.63	1.24-2.12
**Hypnotics and sedatives**	1219	**Overall**	1.07	0.93-1.23	1.06	0.92-1.22
		10->5 years prior to index date	1.03	0.87-1.22	1.02	0.86-1.20
		5->1 years prior to index date	0.90	0.75-1.08	0.89	0.74-1.07
		≤1 year prior to index date	0.84	0.65-1.09	0.83	0.63-1.07
**Antidepressants**	1759	**Overall**	2.45	2.19-2.77	2.46	2.19-2.78
		10->5 years prior to index date	1.44	1.26-1.64	1.43	1.25-1.64
		5->1 years prior to index date	2.29	2.00-2.61	2.27	1.99-2.60
		≤1 year prior to index date	3.63	3.10-4.25	3.62	3.09-4.24
**Other nervous system products**	754	**Overall**	1.22	1.03-1.44	1.22	1.03-1.44
		10->5 years prior to index date	1.23	1.00-1.50	1.22	1.00-1.49
		5->1 years prior to index date	1.20	0.97-1.49	1.20	0.97-1.50
		≤1 year prior to index date	1.17	0.82-1.68	1.18	0.82-1.70

Table S6. Post-hoc analysis - Incidence rate ratios for first-ever medication use in *nervous system* subcategory (compared to never-users)

Nervous system subcategory	Time-interval	Unadjusted		Adjusted	
		IRR	95% CI	IRR	95% CI
**Analgesics**	10->5 years prior to index date	0.95	0.82-1.26	0.95	0.82-1.09
	5->1 years prior to index date	0.91	0.78-1.06	0.91	0.78-1.06
	≤1 year prior to index date	1.29	0.95-1.77	1.25	0.92-1.72
**Antiepileptics**	10->5 years prior to index date	1.20	0.91-1.59	1.21	0.92-1.61
	5->1 years prior to index date	1.01	0.76-1.33	0.99	0.75-1.31
	≤1 year prior to index date	0.88	0.46-1.68	0.87	0.46-1.66
**Antipsychotics**	10->5 years prior to index date	1.33	0.93-1.91	1.33	0.92-1.91
	5->1 years prior to index date	2.95	2.04-4.27	2.84	1.96-4.13
	≤1 year prior to index date	13.66	6.64-28.12	13.37	6.49-27.57
**Anxiolytics**	10->5 years prior to index date	1.23	0.89-1.43	1.10	0.87-1.40
	5->1 years prior to index date	1.28	0.92-1.77	1.25	0.90-1.73
	≤1 year prior to index date	6.13	3.42-10.98	6.12	3.41-10.97
**Hypnotics and sedatives**	10->5 years prior to index date	1.15	0.93-1.42	1.15	0.93-1.41
	5->1 years prior to index date	1.05	0.79-1.39	1.04	0.78-1.37
	≤1 year prior to index date	1.92	1.11-3.32	1.92	1.11-3.33
**Antidepressants**	10->5 years prior to index date	1.46	1.21-1.76	1.45	1.20-1.75
	5->1 years prior to index date	4.17	3.31-5.25	4.09	3.25-5.16
	≤1 year prior to index date	12.81	8.46-19.40	12.73	8.39-19.29
**Other nervous system products**	10->5 years prior to index date	1.12	0.88-1.41	1.11	0.88-1.40
	5->1 years prior to index date	1.15	0.85-1.57	1.15	0.85-1.56
	≤1 year prior to index date	1.32	0.60-2.92	1.35	0.61-2.99

Table S7. Sensitivity analysis by dementia syndrome severity at time of diagnosis – incidence rate ratios by overall categories

Overall category	MCI/MILD DEMENTIA				MODERATE/SEVERE DEMENTIA			
	Unadjusted		Adjusted		Unadjusted		Adjusted	
	IRR	95% CI	IRR	95% CI	IRR	95% CI	IRR	95% CI
**Alimentary tract and metabolism** A	0.86	0.75-0.99	0.87	0.76-1.00	1.33	1.10-1.60	1.29	1.07-1.55
**Blood and blood forming organs** B	1.26	1.08-1.46	1.28	1.01-1.49	1.54	1.28-1.86	1.51	1.25-1.83
**Cardiovascular system** C	1.05	0.91-1.22	1.07	0.92-1.24	1.09	0.90-1.33	1.07	0.88-1.30
**Dermatologicals** D	0.96	0.84-1.11	0.96	0.83-1.10	1.05	0.87-1.26	1.04	0.86-1.26
**Genito urinary system and sex hormones** G	1.14	0.99-1.31	1.13	0.98-1.31	0.91	0.75-1.10	0.92	0.76-1.12
**Systemic hormonal preparations, excluding sex hormones and insulin** H	0.95	0.80-1.12	0.96	0.81-1.13	0.92	0.74-1.13	0.89	0.72-1.10
**Antiinfectives for systemic use** J	1.02	0.84-1.25	1.01	0.83-1.24	0.93	0.72-1.20	0.92	0.71-1.20
**Antineoplastic and immunomodulating agents** L	0.80	0.50-1.29	0.82	0.52-1.31	0.77	0.43-1.37	0.75	0.42-1.35
**Musculo-skeletal system** M	0.97	0.83-1.12	0.98	0.84-1.13	1.03	0.85-1.25	1.01	0.84-1.23
**Nervous system*** N	1.21	1.04-1.41	1.24	1.06-1.44	1.93	1.55-2.41	1.88	1.50-2.35
**Antiparasitic products, insecticides, and repellents** P	0.91	0.77-1.08	0.91	0.77-1.08	0.98	0.79-1.21	0.95	0.76-1.18
**Respiratory system** R	1.08	0.84-1.24	1.08	0.94-1.24	0.90	0.75-1.07	0.89	0.74-1.07
**Sensory organs** S	1.05	0.91-1.20	1.04	0.91-1.19	0.94	0.78-1.12	0.94	0.79-1.17

*Dementia medication (table S1) is omitted in the conditional logistic regression

Table S8. Sensitivity analysis by age at time of diagnosis – incidence rate ratios by overall categories

Overall category	AGE <55 years				AGE ≥55 YEARS			
	Unadjusted		Adjusted		Unadjusted		Adjusted	
	IRR	95% CI	IRR	95% CI	IRR	95% CI	IRR	95% CI
**Alimentary tract and metabolism** A	0.82	0.52-1.29	0.77	0.49-1.23	1.02	0.91-1.15	1.02	0.91-1.14
**Blood and blood forming organs** B	0.98	0.57-1.69	0.90	0.52-1.56	1.39	1.23-1.56	1.39	1.23-1.57
**Cardiovascular system** C	0.89	0.59-1.38	0.85	0.55-1.33	1.08	0.96-1.22	1.09	0.96-1.23
**Dermatologicals** D	0.92	0.59-1.42	0.92	0.59-1.45	0.98	0.89-1.12	0.99	0.89-1.11
**Genito urinary system and sex hormones** G	1.29	0.78-2.11	1.26	0.76-2.09	1.04	0.93-1.17	1.03	0.92-1.16
**Systemic hormonal preparations, excluding sex hormones and insulin** H	0.83	0.46-1.48	0.81	0.45-1.48	0.94	0.82-1.07	0.93	0.82-1.07
**Antiinfectives for systemic use** J	1.34	0.73-2.45	1.20	0.65-2.23	0.96	0.82-1.13	0.95	0.81-1.12
**Antineoplastic and immunomodulating agents** L	0.86	0.18-4.13	0.86	0.18-4.25	0.79	0.53-1.13	0.78	0.53-1.13
**Musculo-skeletal system** M	0.93	0.58-1.48	0.91	0.56-1.47	0.99	0.88-1.12	0.99	0.88-1.12
**Nervous system*** N	1.53	0.93-2.51	1.44	0.87-2.39	1.41	1.24-1.60	1.41	1.24-1.60
**Antiparasitic products, insecticides, and repellents** P	1.08	0.66-1.78	1.00	0.59-1.68	0.93	0.81-1.06	0.92	0.80-1.06
**Respiratory system** R	1.26	0.82-1.95	1.19	0.76-1.86	0.99	0.89.1.11	0.99	0.88-1.11
**Sensory organs** S	0.86	0.56-1.34	0.81	0.51-1.27	1.02	0.91-1.14	1.01	0.90-1.13

*Dementia medication (table S1) is omitted in the conditional logistic regression

Table S9. Sensitivity analysis by sex – incidence rate ratios by overall categories

Overall category	FEMALE				MALE			
	Unadjusted		Adjusted		Unadjusted		Adjusted	
	IRR	95% CI	IRR	95% CI	IRR	95% CI	IRR	95% CI
**Alimentary tract and metabolism**<br>A	1.05	0.91-1.22	1.05	0.91-1.21	0.95	0.80-1.13	0.95	0.80-1.12
**Blood and blood forming organs**<br>B	1.42	1.22-1.66	1.42	1.21-1.66	1.29	1.08-1.54	1.30	1.08-1.55
**Cardiovascular system**<br>C	0.99	0.85-1.15	1.00	0.86-1.17	1.18	0.99-1.41	1.18	0.98-1.41
**Dermatologicals**<br>D	0.95	0.82-1.10	0.95	0.82-1.10	1.41	0.89-1.24	1.04	0.88-1.24
**Genito urinary system and sex hormones**<br>G	1.05	0.91-1.22	1.07	0.92-1.23	1.04	0.87-1.26	1.01	0.84-1.22
**Systemic hormonal preparations, excluding sex hormones and insulin**<br>H	0.95	0.80-1.11	0.94	0.80-1.11	0.91	0.74-1.13	0.91	0.74-1.12
**Antiinfectives for systemic use**<br>J	0.95	0.75-1.20	0.96	0.76-1.21	1.02	0.82-1.27	0.98	0.79-1.22
**Antineoplastic and immunomodulating agents**<br>L	0.85	0.54-1.31	0.85	0.54-1.32	0.68	0.35-1.32	0.67	0.34-1.29
**Musculo-skeletal system**<br>M	0.95	0.81-1.10	0.95	0.81-1.11	1.05	0.88-1.26	1.03	0.86-1.23
**Nervous system***<br>N	1.46	1.23-1.73	1.44	1.22-1.71	1.37	1.15-1.64	1.37	1.14-1.65
**Antiparasitic products, insecticides, and repellents**<br>P	0.99	0.84-1.16	0.98	0.83-1.15	0.85	0.67-1.06	0.83	0.66-1.05
**Respiratory system**<br>R	0.98	0.84-1.13	0.98	0.85-1.13	1.05	0.89-1.25	1.04	0.88-1.24
**Sensory organs**<br>S	1.03	0.89-1.19	1.04	0.90-1.20	0.97	0.82-1.15	0.95	0.80-1.13

*Dementia medication (table S1) is omitted in the conditional logistic regression

Table S10. Sensitivity analysis, censoring MCI

Overall category	MILD DEMENTIA/MODERATE/SEVERE DEMENTIA			
	Unadjusted		Adjusted	
	IRR	95% CI	IRR	95% CI
**Alimentary tract and metabolism** **A**	1.01	0.90-1.13	1.00	0.90-1.12
**Blood and blood forming organs** B	1.38	1.22-1.55	1.38	1.22-1.55
**Cardiovascular system** **C**	1.06	0.94-1.19	1.05	0.93-1.19
**Dermatologicals** D	1.00	0.89-1.12	1.00	0.89-1.12
**Genito urinary system and sex hormones** G	1.02	0.91-1.15	1.02	0.91-1.14
**Systemic hormonal preparations, excluding sex hormones and insulin** H	0.92	0.81-1.06	0.91	0.80-1.04
**Antiinfectives for systemic use** J	0.97	0.83-1.14	0.97	0.82-1.1.3
**Antineoplastic and immunomodulating agents** L	0.80	0.55-1.17	0.80	0.55-1.16
**Musculo-skeletal system** M	0.98	0.87-1.10	0.98	0.87-1.10
**Nervous system*** N	1.42	1.25-1.61	1.41	1.24-1.61
**Antiparasitic products, insecticides, and repellents** P	0.93	0.81-1.06	0.92	0.80-1.06
**Respiratory system** R	0.99	0.88-1.10	0.99	0.88-1.10
**Sensory organs** S	0.99	0.88-1.11	0.99	0.88-1.11

*Dementia medication (table S1) is omitted in the conditional logistic regression

Table S11. Sensitivity analysis, censoring prescription medication use 6 months prior to index date

Overall category	Time-interval	Unadjusted		Adjusted	
		IRR	95% CI	IRR	95% CI
**Alimentary tract and metabolism**	**Overall**	1.01	0.90-1.13	1.04	0.90-1.12
**A**	10->5 years prior to index date	0.98	0.88-1.10	0.98	0.87-1.09
	5->1 years prior to index date	0.96	0.86-1.07	0.96	0.86-1.07
	≤1 year-6 months prior to index date	0.90	0.78-1.4	0.90	0.78-1.04
**Blood and blood forming organs**	**Overall**	1.36	1.21-1.53	1.37	1.24-1.54
B	10->5 years prior to index date	0.99	0.86-1.14	0.99	0.86-1.14
	5->1 years prior to index date	1.28	1.12-1.45	1.27	1.12-1.45
	≤1 year-6 months prior to index date	1.44	1.23-1.67	1.44	1.24-1.68
**Cardiovascular system**	**Overall**	1.07	0.95-1.20	1.07	0.95-1.20
**C**	10->5 years prior to index date	1.04	0.93-1.16	1.05	0.94-1.17
	5->1 years prior to index date	1.06	0.95-1.18	1.07	0.95-1.19
	≤1 year-6 months prior to index date	1.00	0.89-1.14	1.01	0.90-1.15
**Dermatologicals**	**Overall**	0.99	0.89-1.11	0.99	0.89-1.11
D	10->5 years prior to index date	1.06	0.95-1.18	1.06	0.95-1.18
	5->1 years prior to index date	0.89	0.80-0.99	0.89	0.79-0.99
	≤1 year-6 months prior to index date	0.93	0.77-1.13	0.92	0.76-1.12
**Genito urinary system and sex hormones**	**Overall**	1.05	0.94-1.18	1.05	0.93-1.17
G	10->5 years prior to index date	1.06	0.94-1.20	1.06	0.94-1.20
	5->1 years prior to index date	1.05	0.93-1.18	1.04	0.92-1.18
	≤1 year-6 months prior to index date	0.76	0.63-0.92	0.75	0.63-0.91
**Systemic hormonal preparations, excluding sex hor-**	**Overall**	0.93	0.82-1.06	0.93	0.82-1.06
**mones and insulin**	10->5 years prior to index date	1.00	0.87-1.17	1.00	0.86-1.16
H	5->1 years prior to index date	0.93	0.78-1.08	0.91	0.79-1.07
	≤1 year-6 months prior to index date	0.92	0.73-1.16	0.92	0.73-1.16
**Antiinfectives for systemic use**	**Overall**	0.99	0.84-1.16	0.98	0.84-1.14
J	10->5 years prior to index date	1.04	0.92-1.18	1.04	0.91-1.17
	5->1 years prior to index date	0.86	0.77-0.96	0.85	0.76-0.96
	≤1 year-6 months prior to index date	0.99	0.84-1.16	0.99	0.84-1.16
**Antineoplastic and immunomodulating agents**	**Overall**	0.79	0.55-1.14	0.78	0.51-1.13
L	10->5 years prior to index date	0.60	0.38-0.96	0.60	0.38-0.97
	5->1 years prior to index date	1.01	0.65-1.57	1.00	0.65-1.56
	≤1 year-6 months prior to index date	1.00	0.52-1.93	1.00	0.52.1.93
**Musculo-skeletal system**	**Overall**	0.99	0.88-1.11	0.99	0.88-1.11
M	10->5 years prior to index date	1.03	0.92-1.14	1.03	0.92-1.15
	5->1 years prior to index date	0.95	0.85-1.06	0.95	0.85-1.06
	≤1 year-6 months prior to index date	0.81	0.68-0.96	0.80	0.67-0.95

**Nervous system***	**Overall**	1.42	1.25-1.60	1.41	1.25-1.60
N	10->5 years prior to index date	1.18	1.06-1.31	1.17	1.05-1.31
	5->1 years prior to index date	1.21	1.08-1.35	1.20	1.07-1.34
	≤1 year-6 months prior to index date	1.41	1.24-1.60	1.40	1.24-1.59
**Antiparasitic products, insecticides, and repellents**	**Overall**	0.94	0.82-1.07	0.93	0.81-1.06
P	10->5 years prior to index date	1.02	0.87-1.19	1.01	0.87-1.19
	5->1 years prior to index date	0.86	0.72-1.03	0.85	0.71-1.02
	≤1 year-6 months prior to index date	0.89	0.57-1.40	0.88	0.56-1.39
**Respiratory system**	**Overall**	1.01	0.90-1.13	1.01	0.90-1.12
R	10->5 years prior to index date	1.01	0.90-1.13	1.01	0.90-1.13
	5->1 years prior to index date	0.92	0.82-1.03	0.92	0.82-1.03
	≤1 year-6 months prior to index date	0.61	0.51-0.74	0.61	0.51-0.73
**Sensory organs**	**Overall**	1.01	0.90-1.12	1.00	0.90-1-12
S	10->5 years prior to index date	1.12	1.00-1.25	1.12	1.00-1.25
	5->1 years prior to index date	0.87	0.77-0.98	0.87	0.77-0.98
	≤1 year-6 months prior to index date	0.77	0.61-0.97	0.77	0.61-0.97

*Dementia medication (table S1) is omitted in the conditional logistic regression

Supplementary figures

Figure S1. Incidence rate ratios for medication use in *nervous system* subcategory in time intervals (first prescriptions only)

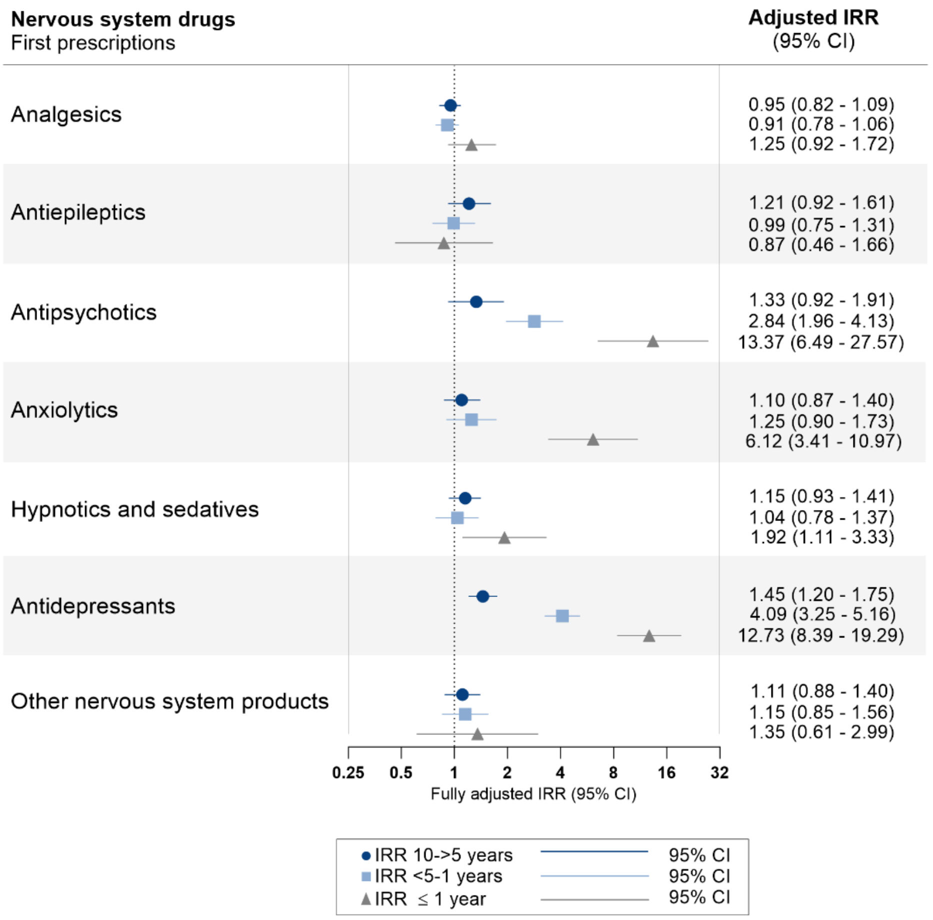

Figure S1 legend: Incidence rate ratios (IRRs) for young onset Alzheimer's disease are plotted by first-ever prescriptions, compared to no prescriptions, in the *nervous system* subcategories in the 10-year retrospective study period and in three time-intervals prior to diagnosis. Conditional logistic regression analyses produced odds ratios, which given the use of incidence-density matching is interpretable as IRRs. For the refence group (dementia-free controls), the IRR is equal to 1 (as indicated by the dotted vertical line). Error bars represent 95% confidence intervals (CI). The IRRs presented are adjusted for age, sex, highest attained educational level at age 40 years (or at time of diagnosis if age at diagnosis <40 years), and civil status at index date. Unadjusted estimates are presented in table S6.

Figure S2. Sensitivity analysis: Incidence rate ratios by overall medication category in the 10-year study period, for the entire study population and divided by disease severity at time of diagnosis.

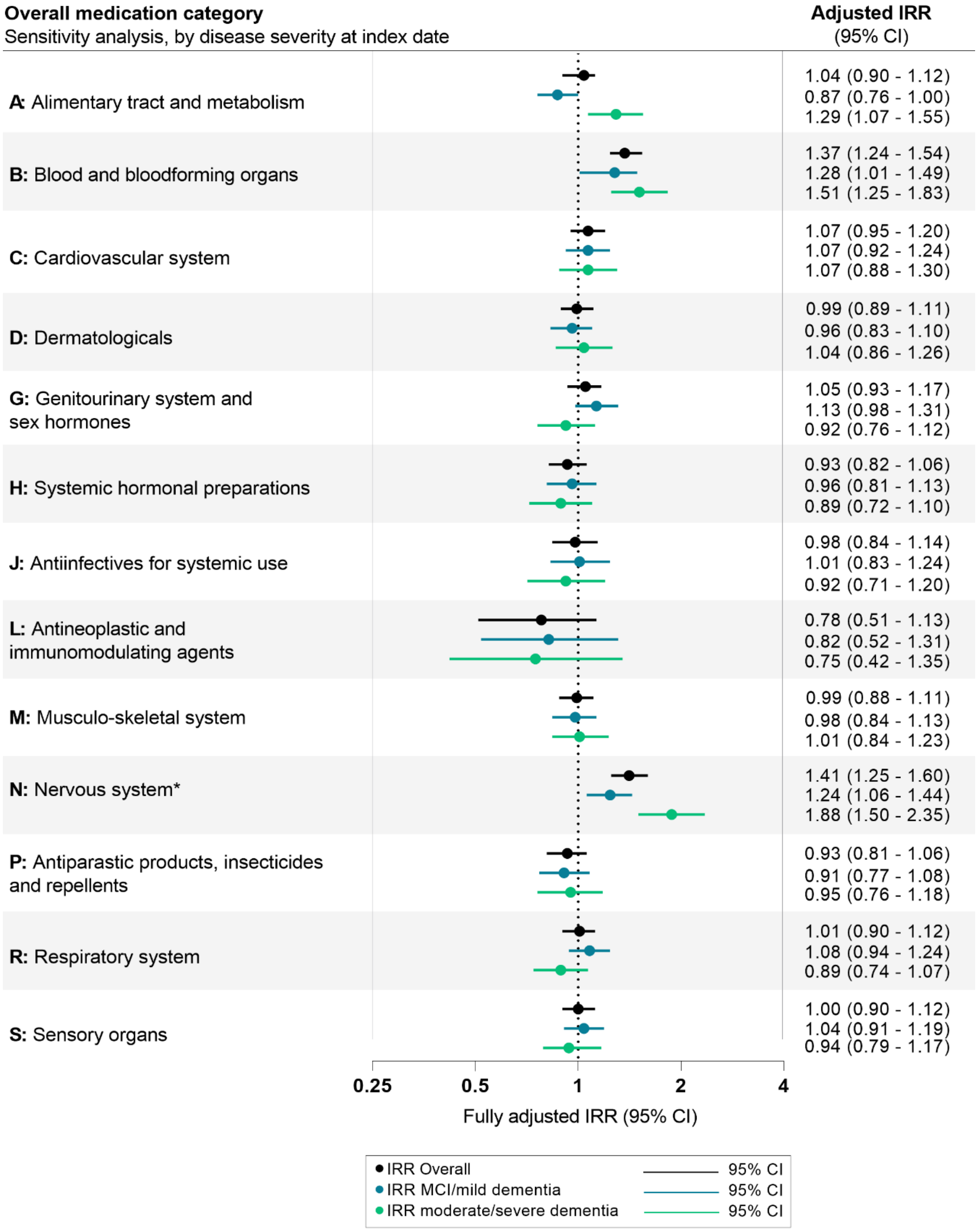

Figure S2 legend: Incidence rate ratios (IRRs) for young onset Alzheimer's disease are plotted by overall medication categories in the 10-year retrospective study period and in three time-intervals prior to diagnosis for the entire study population and divided by disease severity at time of diagnosis. Conditional logistic regression analyses produced odds ratios, which given the use of incidence-density matching is interpretable as IRRs. For the refence group (dementia-free controls), the IRR is equal to 1 (as indicated by the dotted vertical line). Error bars represent 95% confidence intervals (CI). The IRRs presented are adjusted for age, sex, highest attained educational level at age 40 years (or at time of diagnosis if age at diagnosis <40 years), and civil status at index date. Unadjusted estimates are presented in table S7. * Not including dementia medication (see ATC-codes used in table S2).

Figure S3. Sensitivity analysis: Incidence rate ratios by overall medication category in the 10-year study period and in time intervals – for the entire study population and stratified by age.

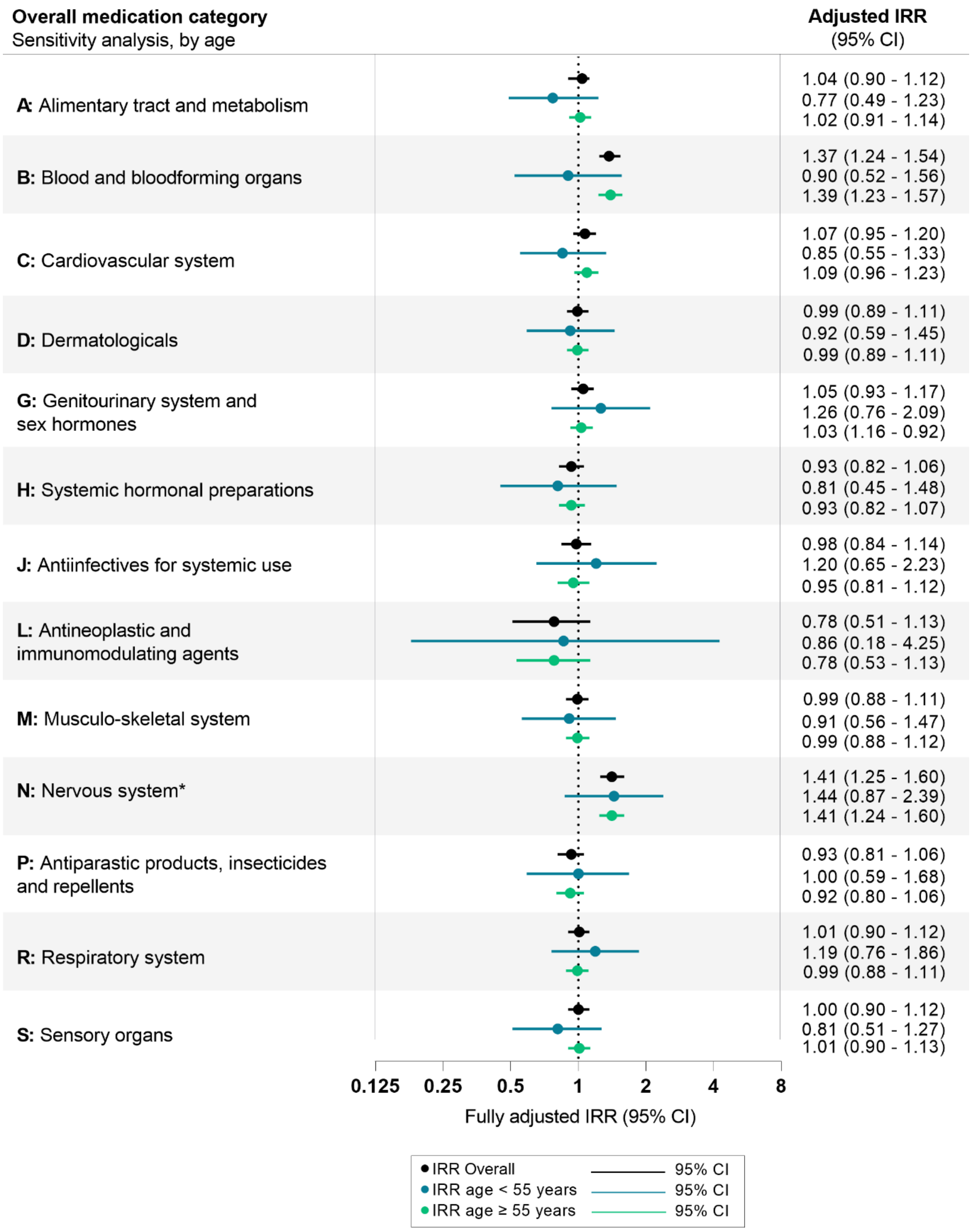

Figure S3 legend: Incidence rate ratios (IRRs) for young onset Alzheimer's disease are plotted by overall medication categories in the 10-year retrospective study period and in three time-intervals prior to diagnosis for the entire study population and stratified by age. Conditional logistic regression analyses produced odds ratios, which given the use of incidence-density matching is interpretable as IRRs. For the refence group (dementia-free controls), the IRR is equal to 1 (as indicated by the dotted vertical line). Error bars represent 95% confidence intervals (CI). The IRRs presented are adjusted for age, sex, highest attained educational level at age 40 years (or at time of diagnosis if age at diagnosis <40 years), and civil status at index date. Unadjusted estimates are presented in table S8. * Not including dementia medication (see ATC-codes used in table S2).

Figure S4. Sensitivity analysis: Incidence rate ratios by overall medication category in the 10-year study period and in time intervals – for the entire study population and divided by sex.

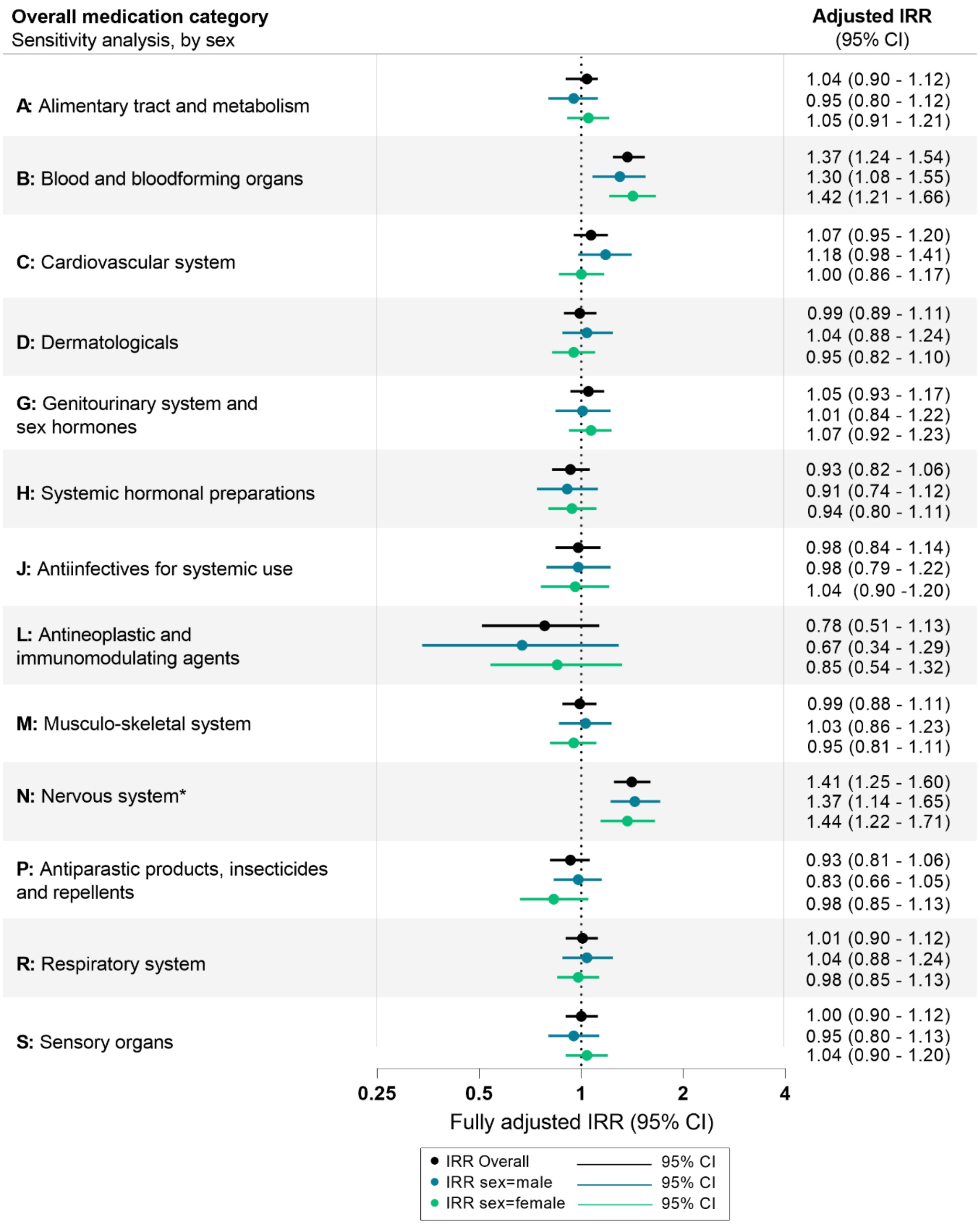

Figure S4 legend: Incidence rate ratios (IRRs) for young onset Alzheimer's disease are plotted by overall medication categories in the 10-year retrospective study period and in three time-intervals prior to diagnosis for the entire study population and divided by sex. Conditional logistic regression analyses produced odds ratios, which given the use of incidence-density matching is interpretable as IRRs. For the refence group (dementia-free controls), the IRR is equal to 1 (as indicated by the dotted vertical line). Error bars represent 95% confidence intervals (CI). The IRRs presented are adjusted for age, highest attained educational level at age 40 years (or at time of diagnosis if age at diagnosis <40 years), and civil status at index date. Unadjusted estimates are presented in table S9. * Not including dementia medication (see ATC-codes used in table S2).
